# Complete genome sequence of *Weissella cibaria* strain CXO-01 isolated from Korean saliva

**DOI:** 10.1128/mra.00151-24

**Published:** 2024-11-22

**Authors:** HyungWoo Jo, Dong-Geol Lee, Hye-Been Kim, Jungeun Kim, Seunghyun Kang, Seyoung Mun, Myong-Hwan Karm, Hyun Jeong Kim

**Affiliations:** 1 COSMAX BTI, R&I Center, Seongnam-si, Gyeonggi-do, South Korea; 2 Department of Microbiology, Dankook University, Cheonan, South Korea; 3 COSMAX NS, INC, Seongnam, South Korea; 4 Center for Bio Medical Engineering Core Facility, Dankook University, Cheonan, South Korea; 5 Department of Dental Anesthesiology, School of Dentistry and Dental Research Institute, Seoul National University, Seoul, South Korea; Department of Biological Sciences, Wellesley College, Wellesley, Massachusetts, USA

**Keywords:** *Weissella cibaria*

## Abstract

We present the genome sequence of *Weissella cibaria* strain CXO-01, isolated from saliva from one Korean individual and kimchi. The genome consisted of one circular chromosome with a length of 2,371,631  bp, three circular plasmids of 20,579 bp, 4,818  bp, and 4,494 in size. Gene prediction revealed 2,305 genes, 2,157 coding DNA sequences.

## ANNOUNCEMENT

*Weissella cibaria* is a Gram-positive, non-spore-forming, non-motile, heterolactic acid-fermenting, catalase-negative bacillus that cannot produce dextran from sucrose (1). Members of *Weissella* are lactic acid bacteria that have been isolated from various sources, including fermented foods such as Korean kimchi, Spanish sausages, Greek salami, human gut, and insect gut (2–5). Recently, certain members of the *Weissella* genus, such as *Weissella cibaria*, *Weissella koreensis*, and *Weissella confusa*, were reported to dominate during the early stages of kimchi fermentation (6, 7). *W. cibaria* has probiotic properties (6, 8, 9). Here, we report the complete genome sequence of *W. cibaria* strain CXO-01 isolated from saliva samples from one Korean individual.

To isolate *W. cibaria* CXO-01, 1 mL of saliva sample was serially diluted using sterilized 0.85% saline solution, and 100 µL of the diluted samples was spread onto an De Man–Rogosa–Sharpe (MRS) agar (Cat. no. 288130, Difco, USA) which was incubated aerobically at 30°C for 72 h. White colonies with varied morphologies were individually separated and streaked on MRS agar to obtain a single colony. The 16S rRNA gene sequences of the isolated strains were identified by Sanger sequencing from Macrogen Co., Ltd. (Seoul, Republic of Korea) and analyzed using an Applied Biosystems 3730xl DNA Analyzer by cycle sequencing using a BigDye Terminator. The similarity of 16S rRNA gene sequences was calculated using the EzBioCloud database (https://www.ezbiocloud.net/identify). The 16S rRNA gene sequences were submitted to the National Center for Biotechnology Information under the GenBank accession number OQ164819.

The genomic DNA of *W. cibaria* CXO-01 was extracted using the Wizard Genomic DNA Purification Kit (Promega, USA) following the manufacturer’s protocol. The quantity and quality of the isolated DNA were determined using a NanoDrop spectrophotometer. DNA shearing was performed using MP Biomedicals FastPrep (Thermo Fisher Scientific Inc, USA) to ensure the optimal DNA size for library construction. Genomic DNA ScreenTape Analysis utilizing TapeStation (Agilent technologies, USA) was used to isolate, size, quantify, and analyze the integrity of the DNA samples.

To sequence the whole genome of *W. cibaria* CXO-01, a SMRTbell library with 20-kb insert size was constructed using the PacBio SMRTbell prep kit 3.0 and analyzed with the Sequel II Bind kit 3.2 and Sequel II DNA internal control complex 3.2 (Pacific Biosciences, USA). The genome was sequenced on a PacBio sequel IIe sequencing platform using single-molecule real-time (SMRT) cell 8 tray and Sequel II sequencing kit 2.0 (Pacific Biosciences, USA). Approximately 0.272 GB of HiFi bases were produced and used for the further steps. A total of 35,963 HiFi reads (average HiFi read length: 6,748 bp; HiFi read length N50: 8,284 bp) of *W. cibaria* CXO-01 were generated. PacBio reads were assembled using the official PacBio software for HiFi microbial genome assembly, Improved Phased Assembler (IPA) v1.8. The IPA v1.8 tool built into SMRT LINK v12 optimizes genome assembly, including *de novo* assembly with Nighthawk, alignment with PanCake, and applications for both circularization and rotation correction(10)(https://github.com/PacificBiosciences/pbipa). Default parameters were used except where otherwise mentioned. The assembly resulted in three contigs, including one circular chromosome of 2,371,631  bp (G + C content, 45.29%; coverage, 160× [based on genome size] [GenBank accession number CP090955.1]), three circular plasmids of 20,579 bp, 4,818  bp, and 4,494 in size (G + C content, 36.8%; coverage, 100×). Gene prediction and annotation using the Prokka Pipeline resulted in 2,305 genes, 2,157 coding DNA sequences (CDSs), 28 rRNAs, 88 tRNAs, and one transfer-messenger (tm) RNA on the chromosome, and 31 genes and 31 CDSs on the three plasmids.

## Data Availability

The associated BioProject and BioSample are PRJNA793913 and SAMN24593066, respectively. The raw PacBio reads can be found under the SRA accession number SRR29429614. The complete genome sequence of *Weissella cibaria* strain CXO-1 was deposited in GenBank under accession numbers CP158359, CP158360, CP158361, and CP158362.

## References

[1] Kang MS, Kim BG, Chung J, Lee HC, Oh JS. 2006. Inhibitory effect of Weissella cibaria isolates on the production of volatile sulphur compounds. J Clin Periodontol 33:226–232. doi:10.1111/j.1600-051X.2006.00893.x 16489950

[2] Kwak S-H, Cho Y-M, Noh G-M, Om A-S. 2014. Cancer preventive potential of kimchi lactic acid bacteria (Weissella cibaria, Lactobacillus plantarum). J Cancer Prev 19:253–258. doi:10.15430/JCP.2014.19.4.253 25574459 PMC4285955

[3] Lee S-H, Ku H-J, Ahn M-J, Hong J-S, Lee SH, Shin H, Lee KC, Lee J-S, Ryu S, Jeon CO, Lee J-H. 2015. Weissella jogaejeotgali sp. nov., isolated from jogae jeotgal, a traditional Korean fermented seafood. Int J Syst Evol Microbiol 65:4674–4681. doi:10.1099/ijsem.0.000631 26410078

[4] Praet J, Meeus I, Cnockaert M, Houf K, Smagghe G, Vandamme P. 2015. Novel lactic acid bacteria isolated from the bumble bee gut: Convivina intestini gen. nov., sp. nov., Lactobacillus bombicola sp. nov., and Weissella bombi sp. nov. Antonie Van Leeuwenhoek 107:1337–1349. doi:10.1007/s10482-015-0429-z 25783976

[5] Collins MD, Samelis J, Metaxopoulos J, Wallbanks S. 1993. Taxonomic studies on some leuconostoc-like organisms from fermented sausages: description of a new genus Weissella for the Leuconostoc paramesenteroides group of species. J Appl Bacteriol 75:595–603. doi:10.1111/j.1365-2672.1993.tb01600.x 8294308

[6] Yu HS, Lee NK, Choi AJ, Choe JS, Bae CH, Paik HD. 2019 a. Anti-Inflammatory potential of probiotic strain Weissella cibaria JW15 isolated from kimchi through regulation of NF-κB and MAPKs pathways in LPS-induced RAW 264.7 cells. J Microbiol Biotechnol 29:1022–1032. doi:10.4014/jmb.1903.03014 31216608

[7] Jeong SE, Chun BH, Kim KH, Park D, Roh SW, Lee SH, Jeon CO. 2018. Genomic and metatranscriptomic analyses of Weissella koreensis reveal its metabolic and fermentative features during kimchi fermentation. Food Microbiol 76:1–10. doi:10.1016/j.fm.2018.04.003 30166128

[8] Sun HY, Kim KP, Bae CH, Choi AJ, Paik HD, Kim IH. 2019. Evaluation of Weissella cibaria JW15 probiotic derived from fermented Korean vegetable product supplementation in diet on performance characteristics in adult beagle dog. Animals (Basel) 9:581. doi:10.3390/ani9080581 31434237 PMC6719065

[9] Yu HS, Lee NK, Choi AJ, Choe JS, Bae CH, Paik HD. 2019 b. Antagonistic and antioxidant effect of probiotic Weissella cibaria JW15. Food Sci Biotechnol 28:851–855. doi:10.1007/s10068-018-0519-6 31093443 PMC6484072

[10] Chin C-S, Alexander DH, Marks P, Klammer AA, Drake J, Heiner C, Clum A, Copeland A, Huddleston J, Eichler EE, Turner SW, Korlach J. 2013. Nonhybrid, finished microbial genome assemblies from long-read SMRT sequencing data. Nat Methods 10:563–569. doi:10.1038/nmeth.2474 23644548

